# Estrogen Promotes Pro-resolving Microglial Behavior and Phagocytic Cell Clearance Through the Actions of Annexin A1

**DOI:** 10.3389/fendo.2019.00420

**Published:** 2019-06-26

**Authors:** Rodrigo Azevedo Loiola, Edward S. Wickstead, Egle Solito, Simon McArthur

**Affiliations:** ^1^John Vane Science Centre, Barts and The London School of Medicine and Dentistry, William Harvey Research Institute, Queen Mary University of London, London, United Kingdom; ^2^Laboratoire de la Barrière Hémato-Encéphalique, Faculty Jean Perrin, EA 2465, Université d'Artois, Arras, France; ^3^School of Life Sciences, College of Liberal Arts & Sciences, University of Westminster, London, United Kingdom; ^4^Barts and The London School of Medicine and Dentistry, Institute of Dentistry, Blizard Institute, Queen Mary University of London, London, United Kingdom; ^5^Dipartimento di Medicina Molecolare e Biotecnologie Mediche, Universitá degli Studi di Napoli Federico II, Naples, Italy

**Keywords:** microglia, estrogen, phagocytosis, resolution, annexin A1

## Abstract

Local production of estrogen rapidly follows brain tissue injury, but the role this hormone plays in regulating the response to neural damage or in the modulation of mediators regulating inflammation is in many ways unclear. Using the murine BV2 microglia model as well as primary microglia from wild-type and annexin A1 (AnxA1) null mice, we have identified two related mechanisms whereby estradiol can modulate microglial behavior in a receptor specific fashion. Firstly, estradiol, via estrogen receptor β (ERβ), enhanced the phagocytic clearance of apoptotic cells, acting through increased production and release of the protein AnxA1. Secondly, stimulation of either ERβ or the G protein coupled estrogen receptor GPER promoted the adoption of an anti-inflammatory/pro-resolving phenotype, an action similarly mediated through AnxA1. Together, these data suggest the hypothesis that locally produced estrogen acts through AnxA1 to exert powerful pro-resolving actions, controlling and limiting brain inflammation and ultimately protecting this highly vulnerable organ. Given the high degree of receptor selectivity in evoking these responses, we suggest that the use of selective estrogen receptor ligands may hold therapeutic promise in the treatment of neuroinflammation, avoiding unwanted generalized effects.

## Introduction

The neuroprotective potential of the steroid hormone estrogen has been the focus of numerous investigations, with epidemiological and animal model studies suggesting it may protect in conditions as diverse as stroke, Alzheimer's disease, Parkinson's disease, and traumatic brain injury (1). Despite this, detailed understanding of the mechanisms underlying its actions in the brain remains elusive. Many studies have focused upon the direct effects of estrogen upon damaged/dying neurones, but it is only relatively recently that the relationship between estrogen and the innate defense mechanisms of the brain, principally astrocytes and microglia, has begun to be addressed (2, 3).

Local production of estrogen by astrocytes is one of the first responses of both male and female brain tissue to injury (4), with this production affording significant protection *in vivo* (5). Dysregulated inflammation can be extremely deleterious for the brain (6), hence studies have focused on suppressive actions of estradiol, revealing its ability to limit microglial iNOS activity (7), and production of reactive oxygen species (8), prostaglandins (9), and inflammatory cytokines (10).

However, it is becoming increasingly clear that in the absence of aggravating factors inflammation is naturally self-limiting, with many classical mediators activating resolving pathways (11). This may have significant consequences for future therapeutic development, as generalized suppression of microglial activity significantly worsens neuronal loss in several animal models (12, 13), indicating the complexity of the neuroinflammatory response. We hypothesized that the actions of estrogen upon microglia are more complex than simply reducing pro-inflammatory mediator production, and that it may actively promote pro-resolving/anti-inflammatory microglial behavior.

Whilst the mechanisms of neuroinflammatory resolution are relatively underexplored, significant efforts have been made to understand the drivers of this process in peripheral inflammation (14). Many mediators have been shown to aid in inflammatory resolution and repair, but a key driver of this process is the glucocorticoid-inducible protein annexin A1 (ANXA1), which we and others have shown to exert numerous pro-resolving/anti-inflammatory effects including promotion of neutrophil endothelial detachment (15) and apoptosis (16), monocyte recruitment (17, 18) and macrophage phagocytosis (19). CNS expression of ANXA1 is primarily localized to endothelial cells (20) and the microglia, where we have previously shown it to be a critical player in microglial efferocytosis (21). Moreover, several studies have shown that ANXA1 expression in immune and other cells can be enhanced by estradiol (22–24), suggesting that the role of this protein in anti-inflammatory/pro-resolving actions of estradiol merits investigation.

Microglial phagocytosis, the removal of potentially damaging threats such as invading pathogens or apoptotic cells, is central to their ability to respond to neuroinflammatory challenge. We therefore investigated the ability of estrogen to regulate microglial phagocytic clearance of apoptotic cells in unstimulated and pro-inflammatory conditions, and the consequences of estrogen treatment upon microglial phenotype, focussing on the central pro-resolving actor annexin A1.

## Materials and Methods

### Drugs

Laboratory reagents and culture media were purchased from Sigma-Aldrich (Poole, UK) unless otherwise stated. The selective estrogen receptor alpha (ERα) agonist 4,4′,4″-(4-propyl-[1H]-pyrazole-1,3,5–triyl)trisphenol (PPT), the selective estrogen receptor beta (ERβ) agonist diarylpropionitrile (DPN), the selective G-protein coupled estrogen receptor (GPER) agonist (±)-1-[(3aR^*^,4S^*^,9bS^*^)-4-(6-Bromo-1,-3-benzodioxol-5-yl)-3a,4,5,9b-tetrahydro-3H-cyclop-enta[c]quinolin-8-yl]-ethanone (G1), the selective ERβ antagonist 4-[2-phenyl-5,7-bis (trifluoromethyl-)pyrazolo[1,5-a]pyrimidin-3-yl] phenol (PHTPP) and the selective GPER antagonist (3aS^*^,4R^*^,9bR^*^)-4-(6-Bromo-1,3-benz-odioxol-5-yl)-3a,4,5,9b-3H-cyclopenta[c]quinolone (G15) were all purchased from Tocris Bioscience, UK.

### Cell Culture

Murine microglial BV2 cells (25) were cultured in RPMI medium supplemented with 5% fetal calf serum, 100 μM non-essential amino acids, 2 mM l-alanyl-glutamine, and 50 μg/ml gentamycin (all ThermoFisher Scientific, Poole, UK) at 37°C in 5% CO_2_. PC12 cells (ATCC) were cultured in RPMI medium supplemented with 10% normal horse serum, 5% fetal calf serum, 100 μM non-essential amino acids, 2 mM l-alanyl-glutamine, and 50 μg/ml gentamycin (all ThermoFisher Scientific, UK) at 37°C in 5% CO_2_.

### Knockdown of AnxA1 Expression

BV2 cells (1 × 10^5^ cells per well) were transfected with shRNA plasmids targeting murine AnxA1 from the MISSION TRC shRNA collection (Sigma-Aldrich, St. Louis, MO, USA), or with an empty plasmid control (termed pKCON). Cells were transfected for 48 h using FuGENE HD (Promega, Madison, Wisconsin, USA) according to the manufacturer's instructions, followed by selection for stable clones using puromycin (Promega, Madison, Wisconsin, USA). Transfection was confirmed by western blot analysis.

### Phagocytosis Assay

Phagocytosis was assessed as detailed previously (21). Briefly, PC12 cells were fluorescently labeled with 5-chloromethylfluorescein diacetate (CMFDA; Thermofisher Scientific, UK) according to the manufacturer's instructions, and were treated overnight with 80 μM 6-hydroxydopamine hydrobromide or 40 μM Na_2_S_2_O_5_ vehicle to induce apoptosis. Labeled apoptotic/non-apoptotic PC12 cells were then co-cultured with previously plated BV2 cells at a ratio of 1:3 BV2:PC12 for 2 h at 37°C under 5% CO_2_. Co-cultures were rapidly washed three times with ice-cold PBS to remove non-phagocytic cells, and cells were either detached by gentle scraping using a rubber policeman for analysis by imaging cytometer, or mounted under Mowiol mounting agent for analysis by immunofluorescence microscopy as described previously (21). Cells were examined using an ImageStream^x^ MKII imaging cytometer and INSPIRE software (Amnis Corporation, Seattle, WA, USA), collecting a total of 10,000 events per treatment. Typical examples of phagocytic and non-phagocytic BV2 cells are presented in Supplementary Figure 1. Imaging cytometer analysis was confirmed by parallel analysis using microscopy. All experiments were performed in triplicate.

### Primary Microglial Cultures

All animal work was performed under the UK Animals (Scientific Procedures) Act, 1986. Primary murine microglial cultures were prepared from 12-week-old female AnxA1 null (26) and C57BL/6 wild-type mice (*n* = 6), according to published protocols (27). Briefly, animals were transcardially perfused with ice-cold heparinised saline under pentobarbital anesthesia and brains were removed. Tissue was cut into ~1 mm^3^ pieces and digested by incubation with papain for 30 min at 37°C (1.5 U/ml, Sigma, UK). Resulting cell suspensions were purified by filtration through 0.45 μm cell strainers and by density gradient separation on a 75%:25%:0% Percoll-PBS gradient (GE Johnson, UK). Following washing in PBS, cells were plated at 1.5 × 10^5^ cells/well in 12-well plates and cultured in DMEM supplemented with 10% FCS, 100 mM non-essential amino acids, 2 mM L-alanyl-glutamine, 50 mg/ml gentamycin (all Life Technologies, UK), and 10 ng/ml M-CSF (ThermoFisher Scientific, UK) at 37°C in 5% CO_2_ for 10 days prior to experimentation. Phagocytosis assays were performed by microscopy as described above, with the additional step that cultures were immunostained for Iba1 to identify microglia as described previously (21).

### Flow Cytometry Analysis

BV2 cells were fixed by incubation in 2% formaldehyde for 10 min prior to labeling for 45 min at 4°C with anti-AnxA1 50 ng/ml (21), mouse anti-human ERα 4 μg/ml (sc-71064, Santa Cruz Biotechnology Inc., Santa Cruz, USA); mouse anti-human ERβ 4 μg/ml (sc-53494, Santa Cruz Biotechnology Inc.); goat anti-mouse GPER 5 μg/ml (AF5534, R&D Systems Inc., Minneapolis, USA), APC-conjugated rat anti-mouse CD40 2.5 μg/ml, PE-conjugated rat anti-mouse CD206 5 μg/ml, or appropriate isotype controls (Catalog Nos. 17-0401-82 and 12-2061-82, respectively, both ThermoFisher Scientific, UK). Cells were then washed in PBS and, where appropriate, incubated at 4°C for 30 min with an AF488-conjugated secondary antibody (AnxA1, ERα, ERβ: goat anti-mouse IgG, GPER chicken anti-goat IgG, both diluted 1:300). Inclusion of 0.025% saponin at all stages of the immunostaining process was used to define total vs. surface AnxA1 expression. In all cases, 20,000 events were acquired using a FACSCalibur flow cytometer (BD Biosciences, Cowley, UK) equipped with a 488 nm argon laser; data was analyzed using FlowJo 8.8.2 software (Treestar Inc., Stanford, CA, USA) with positive events being compared to appropriate secondary antibody or isotype controls.

### Quantitative RT-PCR

Total RNA was extracted using the RNeasy mini kit and genomic DNA was removed by on-column digestion using the RNase-Free DNase set, following the manufacturer's instructions (Qiagen, Crawley, UK). cDNA was synthesized using 1 μg of pooled RNA from at least three replicates using SuperScript III Reverse Transcriptase (ThermoFisher Scientific, UK). Real-time PCR was performed in triplicate, with 200 ng of cDNA per well, 1 μl of primers, and Power SYBR Green PCR master mix (Applied Biosystems, Warrington, UK), using the ABI Prism 7900HT sequence detection system (Applied Biosystems, UK). The following QuantiTect primers (Qiagen, UK) were used: ribosomal protein L32 (Rpl32; QT00131992), AnxA1 (QT00145915). A dissociation step was always included to confirm the absence of unspecific products. Relative expression was calculated as 2^−ΔΔ*CT*^ using Rpl32 as an endogenous control.

### ELISA

TNFα, was assayed by murine-specific sandwich ELISA using commercially available kits, according to the manufacturer's protocols (ThermoFisher Scientific, UK). AnxA1 was assayed using an in-house ELISA. Briefly, a 96-well plate coated with 10 μg/well of a murine monoclonal anti-ANXA1 Ab was blocked with 1% FCS in 50 mM Na_2_CO_3_ and 50 mM NaHCO_3_ (pH 9.6) for 1 h at 37°C, washed, and samples and a standard series of hrANXA1 were then added to the plate in triplicate and incubated for 1 h at 37°C. Wells were washed, and incubated with a rabbit anti-ANXA1 polyclonal Ab, diluted 1:1,000 (Thermofisher Scientific, UK) for 1 h at 37°C. Wells were washed and incubated with HRP-conjugated sheep anti-rabbit polyclonal antiserum (1:500, Bio-Rad Antibodies, UK) for 1 h at 37°C. Plates were washed and incubated with 3,3′,5,5′-tetramethylbenzidine (Sigma-Aldrich, UK) for 10 min at room temperature; reactions were stopped by addition of 0.5 M H_2_SO_4_. A CLARIOstar spectrophotometer (BMG Labtech, Germany) was used to measure absorbance at 450 nm; minimum sensitivity was 1.4 ng/ml. Nitric oxide production was assessed using the Griess reaction for its stable proxy nitrite (28). Briefly, 100 μl/well culture medium was incubated with an equal volume of Griess reagent (1% sulfanilamide, 0.1% naphthylethylenediamine dihydrochloride, 5% phosphoric acid) for 15 min, prior to measurement of absorbance at 540 nm using a CLARIOstar spectrophotometer (BMG Labtech, Germany). Nitrite production was determined by comparison with a NaNO_2_ standard curve; minimum sensitivity was 3 μM.

### Western Blot Analysis

Samples boiled in 6× Laemmli buffer were subjected to standard SDS-PAGE (10%) and electrophoretically blotted onto Immobilon-P polyvinylidene difluoride membranes (Merck, UK). Total protein was quantified using Ponceau S staining (Merck, UK) and membranes were blotted for AnxA1 using an antibody raised against murine AnxA1 (1:1,000; ThermoFisher Scientific, UK) in Tris-buffer saline solution containing 0.1% Tween-20 and 5% (w/v) non-fat dry milk overnight at 4°C. Membranes were washed with Tris-buffer saline solution containing 0.1% Tween-20, and incubated with secondary antibody (horseradish peroxidase–conjugated goat anti-rabbit 1:5,000; ThermoFisher Scientific, UK), for 2 h at room temperature. Proteins were then detected using the enhanced chemiluminescence detection kit and visualized on Hyperfilm (Amersham Biosciences, Amersham, UK). Films were digitized and analyzed using ImageJ 1.51w software (National Institutes of Health).

### Statistical Analysis

All quantified data are derived from at least three independent experiments, performed in triplicate, and are expressed as the mean ± standard error of the mean. Data were analyzed by one- or two-way ANOVA as appropriate, with *post hoc* comparison using Tukey's HSD *t*-test. In all cases, *p* ≤ 0.05 was taken as indicating statistical significance.

## Results

### Estradiol Modulates Microglial Phagocytosis in a Receptor-Specific Manner

**Figure 1:**
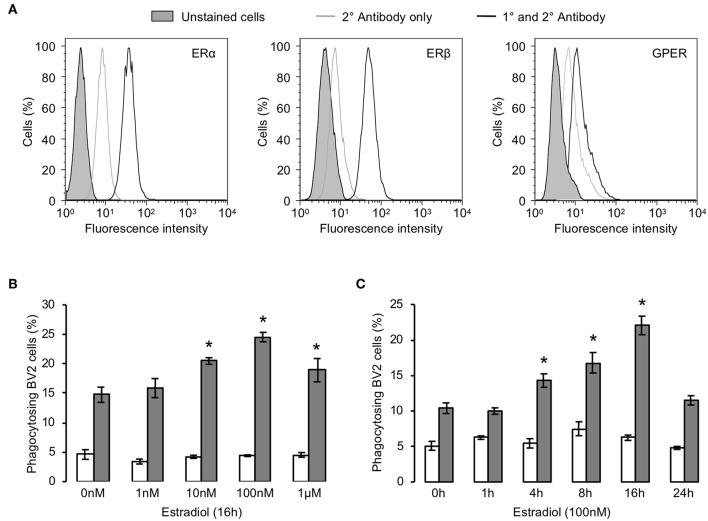
Estrogen promotes microglial phagocytosis of apoptotic cells. **(A)** BV2 cells express all three principal estrogen receptors, ERα, ERβ, and GPER, as determined by flow cytometry. Data are representative histograms of *n* = 3 independent experiments. Exposure of BV2 cells to 17β-estradiol enhances their ability to phagocytose apoptotic (gray) but not non-apoptotic (white) PC12 cells in a **(B)** dose and **(C)** time-dependent manner; data are mean ± sem, *n* = 3, ^*^p < 0.05 vs. untreated controls.

Initial analysis by flow cytometry confirmed the expression of all three principal estrogen receptors (ERα, ERβ, and GPER) are expressed by BV2 microglia (Figure 1A). We then investigated the ability of estrogen to modulate the phagocytosis of apoptotic PC12 cells by BV2 cells. Exposure of BV2 microglia to 17β-estradiol dose- and time-dependently enhanced the phagocytic uptake of apoptotic cells, with greatest impact after 16 h treatment and at 100 nM (Figures 1B,C).

**Figure 2:**
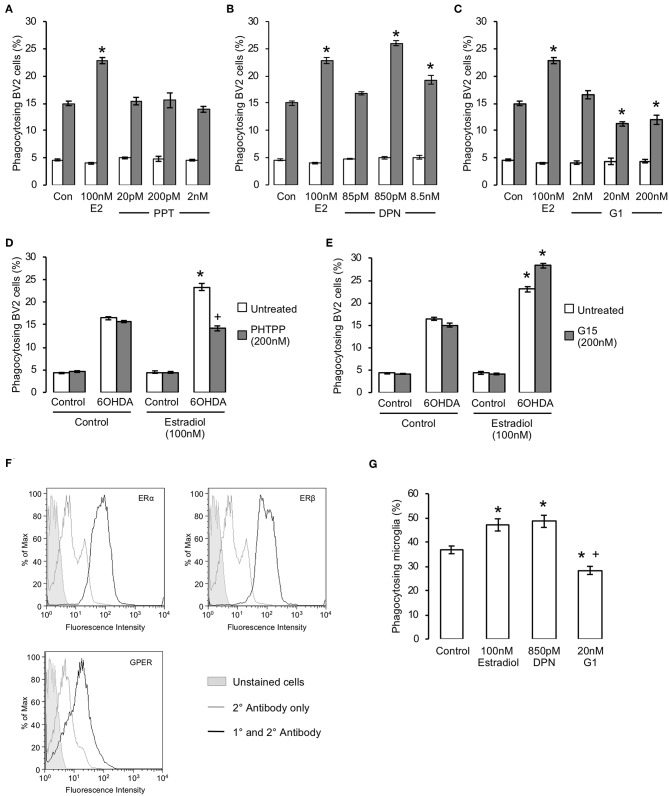
Estrogen has receptor specific effects upon microglial phagocytosis. **(A–C)** Treatment of BV2 cells for 16 h with estradiol (E2) or selective agonists for **(A)** ERα (PPT), **(B)** ERβ (DPN) or **(C)** GPER (G1) revealed receptor dependent effects upon microglial phagocytosis, with activation of ERβ promoting and GPER inhibiting phagocytic activity; ERα activation had no effect on phagocytosis; data are means ± sem, *n* = 3, ^*^p < 0.05 vs. untreated controls. **(D)** Pre-treatment of microglia with the selective ERβ antagonist PHTPP (200 nM, 15 min pre-treatment) prevented the stimulatory effect of estradiol (100 nM, 16 h) upon microglial phagocytosis of apoptotic cells; data are means ± sem, *n* = 3, ^*^p < 0.05 vs. untreated controls, ^+^*p* < 0.05 vs. 17β-estradiol treatment. **(E)** Pre-treatment of microglia with the selective GPER antagonist G1 (200 nM, 15 minute pre-treatment) enhanced the stimulatory effect of estradiol (100 nM, 16 h) upon microglial phagocytosis of apoptotic cells; data are means ± sem, *n* = 3, ^*^p < 0.05 vs. untreated controls, ^+^*p* < 0.05 vs. 17β-estradiol treatment. **(F)** Expression of all three estrogen receptors ERα, ERβ and GPER is detectable in primary murine microglia. **(G)** Treatment of primary murine microglia for 16 h with 17β-estradiol or DPN enhanced phagocytosis of apoptotic cells, whilst treatment with G1 inhibited phagocytosis; data are means ± sem, *n* = 3, ^*^p < 0.05 vs. untreated controls, ^+^*p* < 0.05 vs. 17β-estradiol treatment.

To determine which of the three estrogen receptors were responsible for the pro-phagocytic effects of estradiol, we examined the effect of treatment with specific pharmacological agonists. Treatment of BV2 cells for 16 h with the ERα selective agonist PPT was without effect (Figure 2A) but treatment with the ERβ agonist DPN significantly and dose-dependently enhanced phagocytosis (Figure 2B). In contrast, 16 h exposure to the GPER agonist G1 dose-dependently attenuated the ability of microglia to phagocytose apoptotic cells (Figure 2C). Furthering this analysis, we studied the effects of the ERβ antagonist PHTPP or the GPER antagonist G15 (each administered at their respective IC_50_ values) upon the response of BV2 cells to estradiol. Pre-treatment with either antagonist alone had no effect on phagocytosis of apoptotic cells, but administration of PHTPP almost completely inhibited the pro-phagocytic effect of estradiol (Figure 2D). In contrast, pre-treatment with G15 significantly potentiated apoptotic cell clearance by BV2 cells treated with estradiol (Figure 2E).

To validate the pro-phagocytic effects of estrogen, we examined whether primary microglia derived from adult mouse brain would respond similarly to estradiol or its receptor-specific mimetics. Initial studies confirmed that expression of all three principal estrogen receptor subtypes could be detected in primary murine microglia. (Figure 2F). Primary microglia were significantly more efficient than BV2 cells at phagocytosing apoptotic PC12 cells, but they nonetheless responded in a similar way to estrogen receptor ligand treatment, with estradiol or DPN treatment both potentiating phagocytosis and G1 treatment impairing it (Figure 2G). Together, these data strongly indicate that estradiol exerts dual, and opposing, effects upon microglial phagocytosis acting via both ERβ and GPER.

### Estradiol Promotes Microglial Phagocytosis Through the Mobilization of the Pro-Phagocytic Factor, Annexin A1

**Figure 3:**
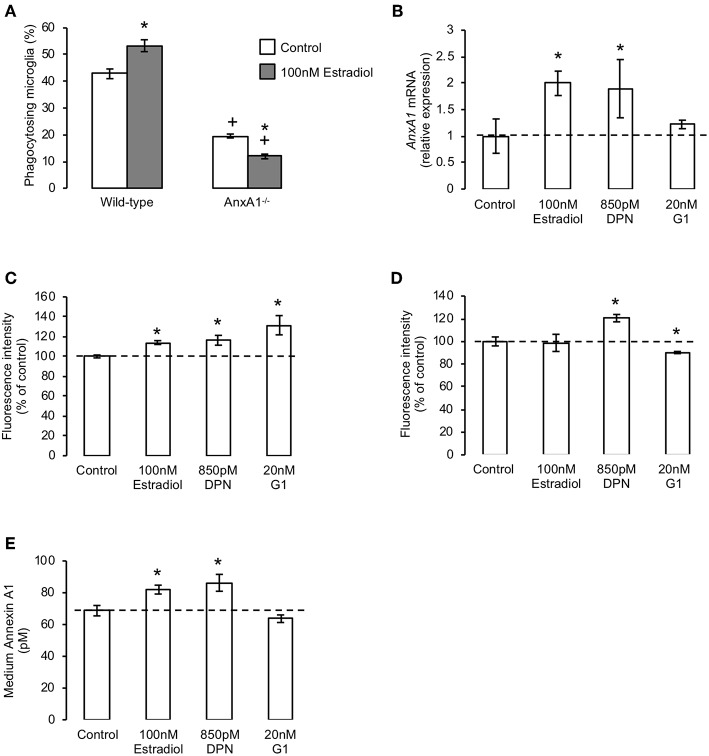
The pro-phagocytic effects of estrogen via ERβ require mobilization of annexin A1. **(A)** Treatment of primary microglia from wild-type mice with 17β-estradiol (100 nM, 16 h) promotes phagocytosis of apoptotic PC12 cells, whereas similar treatment of AnxA1^−/−^ primary microglia causes a significant further inhibition in their phagocytic ability; data are means ± sem, *n* = 3, ^*^p < 0.05 vs. untreated controls, ^+^*p* < 0.05 vs. similarly treated wild-type group. **(B)** Treatment of BV2 cells for 16 h with 17β-estradiol or the ERβ agonist DPN, but not the GPER agonist G1, enhances production of *AnxA1* mRNA; data are means ± sem, *n* = 3, ^*^p < 0.05 vs. untreated controls. **(C)** Treatment of BV2 cells for 16 h with 17β-estradiol, DPN or G1 enhances total cellular AnxA1 content; data are means ± sem, *n* = 3, ^*^p < 0.05 vs. untreated controls. **(D)** Treatment of BV2 cells for 16 h with DPN enhances, whilst similar treatment with G1 reduces, surface expression of AnxA1; data are means ± sem, *n* = 3, ^*^p < 0.05 vs. untreated controls. **(E)** Treatment of BV2 cells for 16 h with 17β-estradiol or DPN but not G1 enhances release of AnxA1 into the culture medium; data are means ± sem, *n* = 3, ^*^p < 0.05 vs. untreated controls.

We have previously shown the microglia-secreted protein annexin A1 (AnxA1) to serve as an important “eat me” signal for phagocytosis following its release from microglia and subsequent binding to exposed phosphatidylserine on the apoptotic cell surface (21). As we and others have shown this protein to mediate some of the actions of estradiol in other contexts (22, 29), we hypothesized that mobilization of AnxA1 may underlie the ERβ-dependent pro-phagocytic effects of estradiol. We therefore compared the effects of the hormone upon phagocytic behavior of primary microglia derived from adult wild-type and AnxA1 null mice. Pre-treatment of primary microglia from wild-type animals with estradiol significantly increased apoptotic cell phagocytosis as expected, but this was not the case in microglia from AnxA1 null mice, where estradiol not only failed to augment microglial phagocytosis, but actually inhibited it (Figure 3A).

Given the importance of AnxA1 secretion from microglia for efficient phagocytosis, we investigated how treatment with estrogen or its receptor-specific mimetics would affect the cellular localization of the protein. Exposure of BV2 cells to either estradiol or the ERβ agonist DPN increased AnxA1 mRNA and total protein content (Figures 3B,C), and in the case of DPN also increased cell surface AnxA1 expression (Figure 3D). Moreover, both ligands significantly induced protein release into the culture medium (Figure 3E). In contrast, treatment with the GPER agonist G1 had no significant effect upon AnxA1 mRNA (Figure 3B) or secreted protein (Figure 3E), but significantly enhanced total AnxA1 content (Figure 3C) whilst decreasing cell surface protein (Figure 3D), strongly suggesting increased intracellular accumulation of the protein.

### Estradiol Regulates Inflammatory Microglial Activation, Promoting Resolution

**Figure 4:**
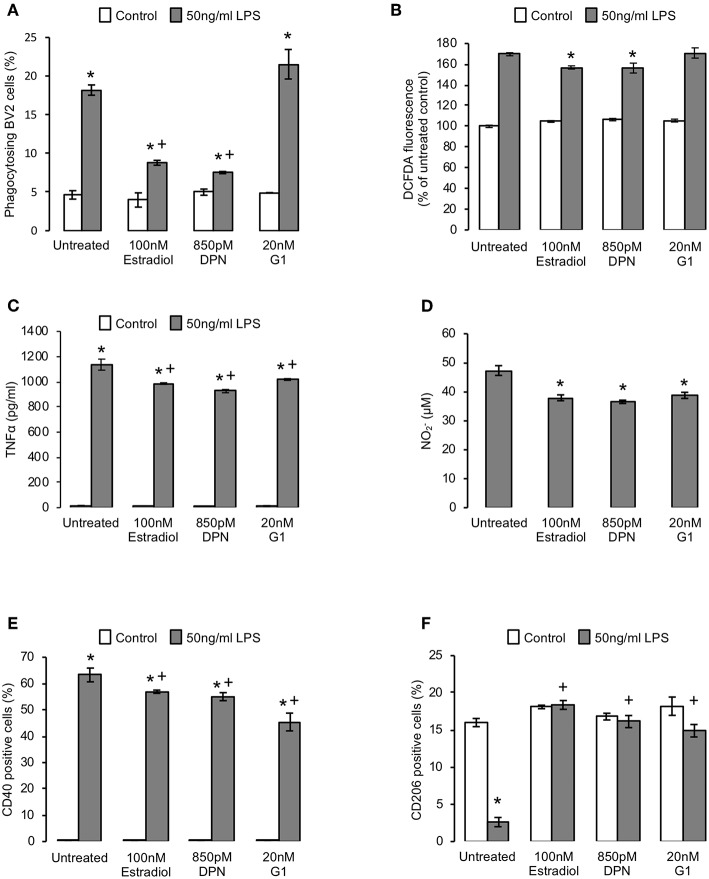
Estrogen promotes an anti-inflammatory microglial phenotype. **(A)** Pre-treatment of BV2 cells for 2 h with 50 ng/ml bacterial lipopolysaccharide (LPS) induces the phagocytosis of non-apoptotic PC12 cells, an effect reversed by subsequent treatment (16 h) with either 17β-estradiol or DPN, but not G1; data are means ± sem, *n* = 3, ^*^p < 0.05 vs. respective control cells, ^+^*p* < 0.05 vs. LPS treatment alone. **(B)** Treatment of BV2 cells with 17β-estradiol or DPN but not G1 (16 h) suppresses LPS-induced reactive oxygen species production (2 h pre-treatment); data are means ± sem, *n* = 3, ^*^p < 0.05 vs. LPS-treated controls. **(C)** Treatment of BV2 cells (16 h) with 17β-estradiol or DPN but not G1 (16 h) limits LPS-induced production of the pro-inflammatory cytokine TNFα (2 h pre-treatment); data are means ± sem, *n* = 3, ^*^p < 0.05 vs. respective control cells, ^+^*p* < 0.05 vs. LPS treatment alone. **(D)** Treatment of BV2 cells with 17β-estradiol, DPN or G1 (16 h) suppresses LPS-induced production of nitrite (2 h pre-treatment; baseline nitrite production was below detection limits); data are means ± sem, *n* = 3, ^*^p < 0.05 vs. LPS-treated control cells. **(E)** Treatment of BV2 cells with 17β-estradiol, DPN or G1 (16 h) suppresses LPS-induced surface expression of the pro-inflammatory marker CD40 (2 h pre-treatment); data are means ± sem, *n* = 3, ^*^p < 0.05 vs. untreated control cells, ^+^*p* < 0.05 vs. LPS-treated control cells. **(F)** Treatment of BV2 cells with 17β-estradiol, DPN or G1 (16 h) reverses the LPS-induced loss of surface expression of the anti-inflammatory marker CD206 (2 h pre-treatment); data are means ± sem, *n* = 3, ^*^p < 0.05 vs. respective control cells, ^+^*p* < 0.05 vs. LPS treatment alone.

Outside of development and certain defined areas of the brain, non-phlogistic neuronal apoptosis is rare, with cell death being more commonly associated with neuroinflammation and disease (30). We therefore investigated the effects of estradiol upon microglia under inflammatory conditions, modeled by exposure to bacterial endotoxin (21, 31). As previously described (21), pre-exposure of BV2 cells to 50 ng/ml LPS for 18 h significantly increased the inappropriate phagocytosis of non-apoptotic cells, but this was markedly attenuated by subsequent (2 h after LPS stimulation) addition of estradiol or DPN, but not G1, indicative of an ERβ-mediated action (Figure 4A). Similarly, LPS-induced production of intracellular reactive oxygen species was reduced by treatment with estradiol and DPN, but not G1 (Figure 4B). In contrast, estradiol, DPN and G1 were all able to attenuate LPS-induced production of the inflammatory mediators TNFα and nitric oxide (Figures 4C,D). Characterization of BV2 cell surface markers of inflammatory phenotype revealed that treatment with estradiol, DPN or G1 could attenuate LPS-induced expression of the pro-inflammatory marker CD40 (Figure 4E, typical profiles Supplementary Figure 2A) and prevent LPS-induced suppression of the anti-inflammatory marker CD206 (Figure 4F, typical profiles Supplementary Figure 2B). Together these data suggest that estrogen receptor activation could limit the pro-inflammatory stimulation of microglia, with significant roles for both ERβ and G1.

**Figure 5:**
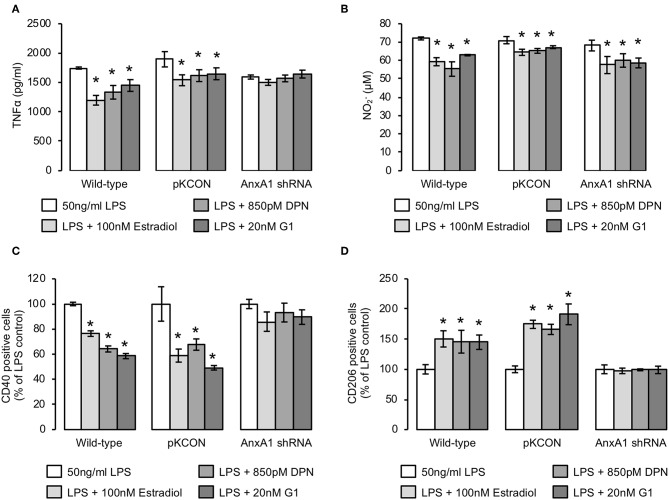
The anti-inflammatory effects of estrogen are largely dependent upon AnxA1 expression. **(A)** Stable transfection of BV2 cells with an shRNA sequence targeting AnxA1, but not with an empty plasmid control (pKCON), inhibits the ability of estradiol, DPN or G1 (16 h) to suppress LPS-induced TNFα production (2 h pre-treatment); data are means ± sem, *n* = 3, ^*^p < 0.05 vs. LPS-treated control cells. **(B)** Neither stable transfection of BV2 cells with an empty plasmid control (pKCON) nor with an shRNA sequence targeting AnxA1 affects the ability of estradiol, DPN or G1 (16 h) to suppress LPS-induced nitrite production (2 h pre-treatment); data are means ± sem, *n* = 3, ^*^p < 0.05 vs. LPS-treated control cells. **(C)** Stable transfection of BV2 cells with an shRNA sequence targeting AnxA1, but not with an empty plasmid control (pKCON), inhibits the ability of estradiol, DPN or G1 (16 h) to suppress LPS-induced surface expression of the pro-inflammatory marker CD40 (2 h pre-treatment); data are means ± sem, *n* = 3, ^*^p < 0.05 vs. LPS-treated control cells. **(D)** Stable transfection of BV2 cells with an shRNA sequence targeting AnxA1, but not with an empty plasmid control (pKCON), inhibits the ability of estradiol, DPN or G1 (16 h) to reverse LPS-suppressed expression of the anti-inflammatory marker CD206 (2 h pre-treatment); data are means ± sem, *n* = 3, ^*^p < 0.05 vs. LPS-treated control cells.

Given the major pro-resolving and anti-inflammatory functions of AnxA1 identified from studies of other immune cells (32), and our previous detection of a key mediating role for this protein in estrogen-induced phagocytosis, we hypothesized that it may similarly mediate the anti-inflammatory actions of estrogen and its receptor mimetics. We therefore stably transfected BV2 cells with a lentiviral vector bearing an shRNA sequence targeting AnxA1 to reduce expression of the protein (Supplementary Figure 3) and investigated whether the anti-inflammatory effects of estrogen or its mimetics were maintained. Knock-down of AnxA1 in this way significantly impaired the ability of estradiol, DPN, or G1 to reverse LPS-induced TNFα release (Figure 5A), but did not affect the inhibitory effects of estradiol, DPN, or G1 on LPS-induced nitric oxide release (Figure 5B). Suppression of AnxA1 expression did however, reverse the effects of estradiol, DPN, or G1 on both CD40 (Figure 5C) and CD206 (Figure 5D) expression, confirming an important role for AnxA1 in mediating many, although not all, of the anti-inflammatory actions of estrogen.

## Discussion

Estrogen is increasingly recognized as a powerful modulator of immune cell activity, able to regulate cells of both the innate (33) and adaptive (2) arms of the immune system, actions which contribute to the sex differences commonly seen in inflammatory disorders. Inflammatory diseases of the CNS, including such major conditions as Alzheimer's disease, Parkinson's disease, and multiple sclerosis, have similarly been shown to be sexually dimorphic in incidence and/or symptom severity (34, 35). Whilst multiple factors undoubtedly contribute to these sex differences, direct neuroprotective effects of estrogen are strongly supported (36, 37). For many years, studies have focussed on the directly neuroprotective actions of estrogen upon neurones (38), but more recent attention has been given to the modulatory effects of the hormone upon microglial behavior, given the importance of these cells in many neurological disorders (31, 39–41). In this study, we have identified the ability of estrogen to modulate a key aspect of microglial function in disease, the removal of apoptotic cells. We describe a clear, receptor-specific, modulatory action of the principal estrogen 17β-estradiol upon microglial clearance of apoptotic cells, and highlight the ability of the steroid to suppress inflammatory microglial activation. Moreover, we reveal a central role for the powerful pro-resolving protein AnxA1 in mediating both the pro-efferocytosis and anti-inflammatory effects of estrogen upon microglia.

Clearance of apoptotic cells by phagocytosis is critical for the maintenance of healthy tissue, as apoptotic cells that are not removed will progress to secondary necrosis and become significant inflammatory foci themselves (42). Microglia are the principal phagocytes of the CNS and play a central role in this process, a particularly important action given the susceptibility of neurones to inflammatory damage (43). Our data reveal estrogen to strongly potentiate microglial phagocytosis of apoptotic cells, acting through induction of AnxA1 expression and secretion. We have previously shown AnxA1 to induce a non-phlogistic phenotype in microglia post-phagocytosis (21), strongly suggesting that its modulation by estrogen is a significant component of the hormone's neuroprotective effects. Previous work has suggested that estrogen can promote phagocytosis by peripheral macrophages (44, 45), acting primarily through ERα. In contrast, whilst we identified ERα expression in microglia, activation of this receptor with the specific ERα agonist PPT had little impact on phagocytosis, with much clearer roles for the alternative estrogen receptors ERβ and GPER.

Whilst the overall effect of 17β-estradiol was to stimulate microglial phagocytosis, pharmacological analysis of the specific receptors involved in this process revealed an intriguing distinction between ERβ and GPER, with ERβ acting to promote phagocytosis and GPER inhibiting it. The mechanism underlying this effect appears to revolve around differential effects upon AnxA1 localization; ERβ activation increased AnxA1 synthesis and release, whilst GPER stimulation led to intracellular AnxA1 accumulation and a reduction in secreted protein. The pro-phagocytic actions of AnxA1 are dependent upon its secretion from microglia and consequent binding to phosphatidylserine on apoptotic target cells (21), hence the negative effects of GPER stimulation upon AnxA1 release presumably underlies the effects of this receptor upon phagocytosis. Interestingly, this dichotomy of effect was not replicated in the effects of estrogen receptor activation upon microglial phenotype, with both ERβ and GPER selective ligands being equipotent in ameliorating LPS-induced pro-inflammatory signs. This finding accords well with published studies that show exogenous administration of the GPER agonist G1 to prevent inflammatory microglial activation *in vivo* (46–48), and that identify a mediatory role for GPER in the anti-inflammatory effects of estrogen in cerebral ischaemia (49). These anti-inflammatory effects of estrogen or its receptor-specific mimetics again appeared to be mediated in large part through AnxA1, with microglia stably bearing AnxA1-targeting shRNA sequences showing a clear impairment in many of the anti-inflammatory actions of estrogen and its mimetics.

Our findings reinforce the importance of AnxA1 in the nervous system response to injury (50), with microglia lacking AnxA1 being severely impaired in their phagocytic ability. Moreover, such microglia show a markedly altered response to estradiol with exposure to the hormone actually inhibiting phagocytosis, and inducing only limited anti-inflammatory actions. These data thus reinforce the complexity of the systems that have evolved to regulate microglial function. Moreover, this work highlights the potent regulatory action of estrogen upon AnxA1 expression and activity (22, 23), and extends this role to the cells of the CNS. Thus, complementing its long-studied role as a mediator of glucocorticoid immunomodulatory action (32), our results further emphasize the importance of AnxA1 in the anti-inflammatory effects of femalere sex hormones.

Local up-regulation of aromatase expression and consequent estrogen production is a key part of the response to CNS injury (4, 5, 51), acting to limit damage and preserve tissue integrity. Where and how estrogen exerts its protective effects is less clear however, but blockade of estrogen production by genetic deletion of aromatase leads to significantly enhanced post-injury gliosis (52). The role of microglia in such injury-induced gliosis is complex, as these cells show dramatic and dynamic changes in phenotype (53), and have the potential to be both harmful and protective to surrounding neurones (54). Our findings suggest that a major action of estrogen upon microglia is to promote their more protective functions, enhancing the efferocytosis of damaged/dying cells and limiting expression of pro-inflammatory features. These actions are supported by numerous studies showing exogenous estrogen to be protective in models of neuroinflammatory/neurotoxic damage (37, 55), and particularly by previous work identifying estrogen as an anti-inflammatory modifier of microglial phenotype (56–62). Together with these reports, our study places estrogen as a significant pro-resolving mediator within the brain, acting to stimulate the removal of cellular debris post-injury and to promote an anti-inflammatory environment, highlighting its role as a major part of the brain's endogenous defense mechanisms.

## Data Availability

No datasets were generated or analyzed during the current study.

## Author Contributions

RL, EW, and SM performed experiments and analysis. SM conceived and designed the study. ES produced AnxA1 shRNA clones and provided valuable insight and advice throughout the project. All authors contributed to the writing of the final manuscript.

### Conflict of Interest Statement

The authors declare that the research was conducted in the absence of any commercial or financial relationships that could be construed as a potential conflict of interest.
