# Supplementary material for: Estrogen Promotes Pro-resolving Microglial Behavior and Phagocytic Cell Clearance Through the Actions of Annexin A1
Source: Front Endocrinol (Lausanne). 2019 Jun 26;10:420. doi: 10.3389/fendo.2019.00420 (PMC6607409; doi:10.3389/fendo.2019.00420)

Supplemental Figure Legends

Supplemental Figure 1: Typical flow cytometry profiles for phagocytosing BV2 cells, shown as A) histograms indicating BV2 cell phagocytosis in control conditions and following treatment with estradiol (100nM, 16h), and B) ImageStream^x MKII imaging cytometer images of BV2 cells exposed to CMFDA-labelled apoptotic PC12 cells, identifying typical cells that were positive and negative for phagocytosis.

Supplementary Figure 2: Typical surface expression profiles for A) CD40 and B) CD206 on BV2 cells treated for 16h with estradiol (100nM), DPN (850pM) or G1 (20nM) ± 2h prior treatment with LPS (50ng/ml).

Supplementary Figure 3: Western blot analysis of AnxA1 expression in three independent samples of wild-type BV2 cells or BV2 cells stably transfected with an empty control plasmid (pKCON) or the same plasmid bearing an shRNA sequence for AnxA1. Comparison is made with total loaded protein, as visualised by Ponceau S staining.

Supplemental Figure 1

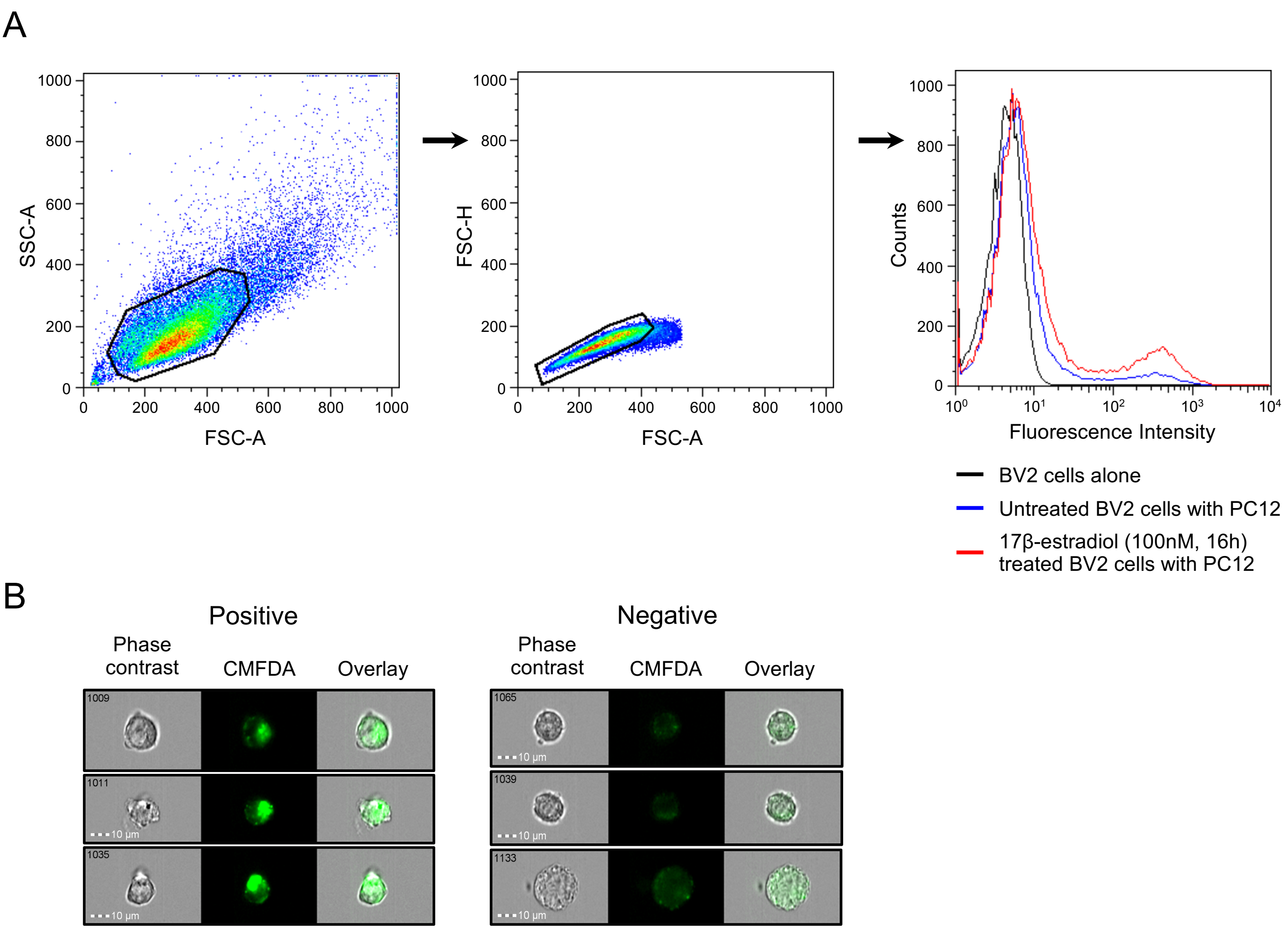

Supplemental Figure 2

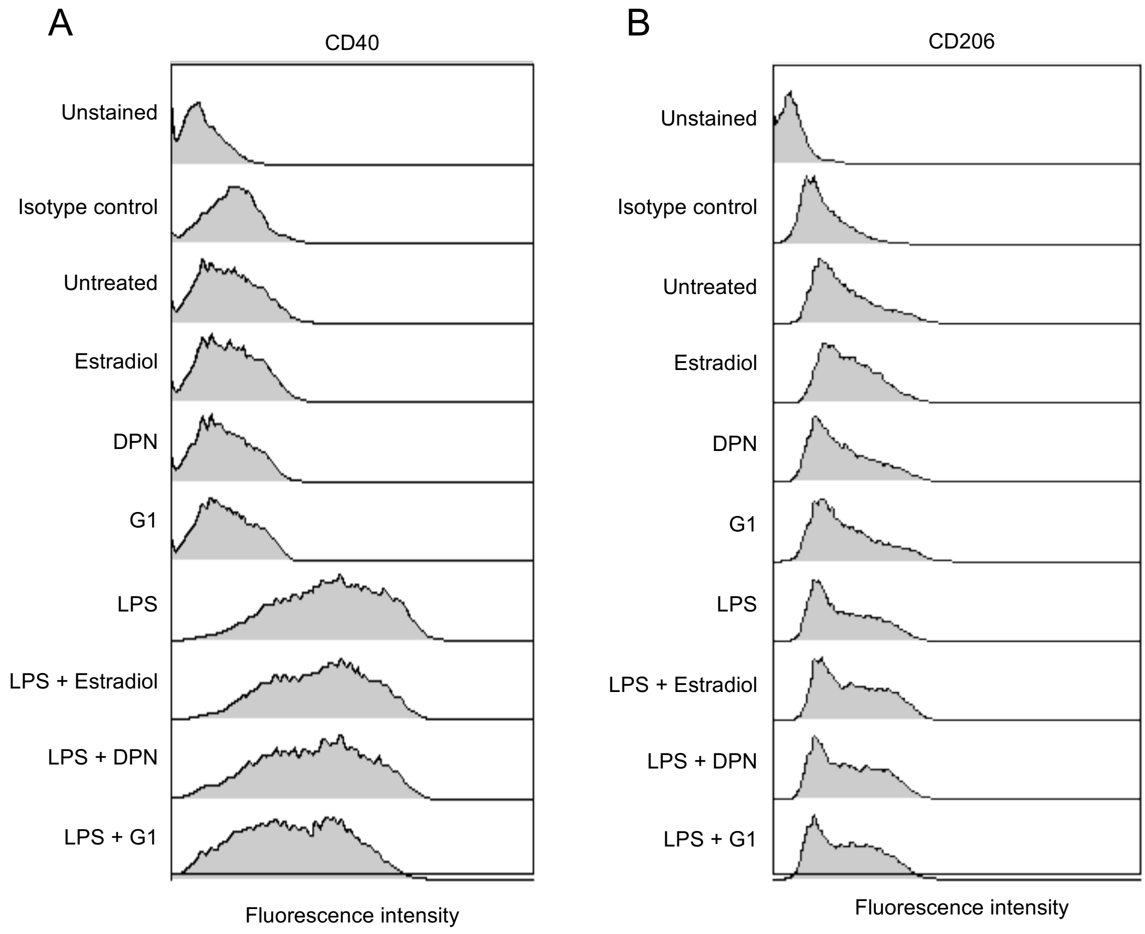

Supplemental Figure 3

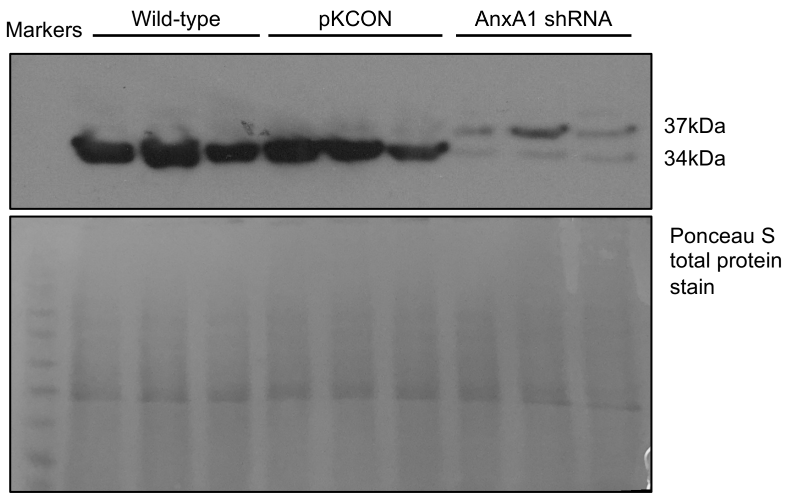

Supplement: Supplementary file 1 [file Data_Sheet_1.PDF]
